# 

**Published:** 1887-06

**Authors:** 


					﻿NOTICE.
All articles for publication, books for review, business communications, etc.,
must be sent to Editors, 1123 Spruce Street, Philadelphia.
Subscribers will please notice that this Journal will be eent until arrears
are paid and it is ordered discontinued.
All subscriptions are due in advance; subscribers will therefore
please forward their subscriptions at once; let each subscriber see
that his account is settled promptly.
Subscribers are respectfully requested to make all checks
and money-orders payable to THE HOMOEOPATHIC
PHYSICIAN, and not to either of the Editors.
				

